# Neurobiology of Anxiety and Depression in CP/CPPS: A Narrative Review of Underlying Mechanisms

**DOI:** 10.3390/neurolint18040069

**Published:** 2026-04-13

**Authors:** Neriman Ezgin, Nikola Šutulović, Emilija Djurić, Slaviša Milošević, Milena Vesković, Dušan Mladenović, Aleksandra Rašić-Marković, Olivera Stanojlović, Dragan Hrnčić

**Affiliations:** 1 Institute of Medical Physiology “Richard Burian”, Faculty of Medicine, University of Belgrade, 11000 Belgrade, Serbia; nerimnezgn@gmail.com (N.E.); nikola.sutulovic@med.bg.ac.rs (N.Š.); emilija.random1@gmail.com (E.D.); allerasic@gmail.com (A.R.-M.); ostanoj@gmail.com (O.S.); 2 Department of Biotechnology, Institute of Natural and Applied Sciences, Cukurova University, Adana 01330, Turkey; 3 Faculty of Sciences and Mathematics, University of Pristina in Kosovska Mitrovica, 38220 Kosovska Mitrovica, Serbia; slavisa.milosevic@pr.ac.rs; 4 Institute of Pathophysiology “Ljubodrag Buba Mihailovic”, Faculty of Medicine, University of Belgrade, 11000 Belgrade, Serbia; msmilenastankovic@gmail.com (M.V.); dusan.mladenovic@med.bg.ac.rs (D.M.)

**Keywords:** prostatitis, chronic pelvic pain, mental health, anxiety, depression, neuroinflammation, neurotransmitters

## Abstract

Chronic prostatitis/chronic pelvic pain syndrome (CP/CPPS) is a prevalent urological disorder characterized by persistent pelvic pain, urinary symptoms, and significant impact on quality of life. In addition to its clinical symptoms, CP/CPPS is frequently associated with psychiatric comorbidities, such as anxiety and depression, indicating complex neurobiological mechanisms. This review explores the mechanisms linking CP/CPPS with affective disorders, emphasizing central nervous system alterations, dysregulation of the hypothalamic–pituitary–adrenal (HPA) axis, and neuroimmune interactions. Evidence in-dicates that central sensitization, microglial and astrocytic activation, and elevated proinflammatory cytokines (IL-1β, IL-6, TNF-α) contribute to maladaptive painemotion network interactions. Additionally, dysregulation of hormones and neurotransmitters may exacerbate both pain perception and mood disorders. Psychosocial factors, including stress, coping strategies, and cognitive-emotional processes, further modulate symptom severity and treatment outcomes, highlighting the importance of a biopsychosocial approach. Gaining a deeper understanding of the neurobiological and psychosocial mechanisms behind anxiety and depression in CP/CPPS can lead to more effective, multidimensional management strategies and enhance patient-centered care.

## 1. Introduction

Chronic prostatitis/chronic pelvic pain syndrome (CP/CPPS) is highly prevalent and clinically heterogeneous urological disorder affecting men. It is characterized by persistent pelvic pain and amplified nociceptive signaling, lower urinary tract symptoms, and varying degree of sexual dysfunction. CP/CPPS imposes substantial individual but also public health burdens since it affects up to 15% of adult males worldwide [1,2,3]. Moreover, CP/CPPS symptoms extend beyond urogenital complaints, frequently co-occurring with brain-related comorbidities in domain of mental health, i.e., psychological and psychiatric one. Anxiety and depression are at the forefront with prevalence approaching 50% in pa-tients with CP/CPPS [4,5]. On the other hand, anxiety and depression as comorbidities significantly impact quality of life by affecting emotional, cognitive, and psychological functioning [6,7,8]. Effective management of CP/CPPS needs to be personalized and multi-faceted. It frequently combines standard pharmacological treatment with non-pharmacological strategies such as neuromodulation techniques and psychological therapies. Despite clear advancements in therapeutic methods, many patients still lack adequate control of symptomss, highlighting the necessity for further improvements.

Although the link between CP/CPPS and mental health comorbidities have been proven in several animal models [9] and confirmed in recent systematic review [10], the neurobiological mechanisms underlying this relationship are still under intensive investigation. In the last decade, there has been significant progress in understanding the mechanisms linking CP/CPPS with anxiety and depression, which have not been comprehensively reviewed until now. In fact, research conducted on experimental models of CP/CPPS has given us important insights [11,12].

Considering that these multidimensional and complex interactions could be of potential interest for development of novel therapeutic approaches, this review comprehensively examines recent molecular, neurobiological, endocrine, and psychosocial underpinnings of anxiety and depression in CP/CPPS. Through this integrative approach, the review provides an integrated framework for understanding the complex psychopathology of the syndrome.

To address this complexity, the present review adopts an integrative mechanistic framework that links peripheral and central processes underlying CP/CPPS and its psychiatric comorbidities. Specifically, we conceptualize the disorder as a multilevel cascade beginning with peripheral immune and inflammatory triggers, which initiate neuroimmune signaling through glial activation and cytokine release. These processes subsequently influence central neurochemical and neuroendocrine systems, including neurotransmitter imbalance and hypothalamic–pituitary–adrenal (HPA) axis dysregulation. Such alterations contribute to dysfunction within key brain circuits involved in pain and emotion regulation, particularly the anterior cingulate cortex, prefrontal cortex, amygdala, and hippocampus. Ultimately, these interconnected mechanisms manifest as behavioral and psychosocial outcomes, including chronic pain, anxiety, depression, and reduced quality of life. By organizing the available evidence within this hierarchical framework, the present review aims to provide a more coherent understanding of the neurobiological basis of anxiety and depression in CP/CPPS and to highlight potential targets for integrated therapeutic strategies.

## 2. Mechanisms of Anxiety and Depression in CP/CPPS

The National Institutes of Health (NIH) classification categorizes prostatitis into four syndromes: acute bacterial prostatitis (type I), chronic bacterial prostatitis (type II), CP/CPPS (type III), and asymptomatic inflammatory prostatitis (type IV). CP/CPPS is further subdivided into inflammatory (IIIa) and non-inflammatory (IIIb) forms based on the presence or absence of leukocytes in expressed prostatic secretions, semen, or post-massage urine. CP/CPPS is not associated with identifiable uropathogenic infection, distinguishing it from bacterial prostatitis and supporting its classification as a chronic pain syndrome with complex underlying mechanisms. Its etiology is considered multifactorial, with proposed initiating mechanisms including: (i) a prior transient infection leading to persistent immune activation. CP/CPPS is often considered a sterile, inflammatory condition, but evidence suggests a post-infectious mechanism, where an initial infection (e.g., bacterial) triggers persistent inflammation or autoimmune responses (cross-reactive antibodies) that remain after the infection is cleared; (ii) autoimmune responses driven by molecular mimicry or loss of tolerance to prostate-specific antigens. in addition to innate and adaptive immune activation, an autoimmune component has been proposed. Experimental and clinical studies have demonstrated T-cell reactivity to prostate-specific antigens, suggesting a breakdown of immune tolerance and the potential involvement of autoimmune mechanisms, possibly triggered by prior infection or antigenic mimicry, and (iii) sterile inflammation arising from neurogenic or stress-related mechanisms. These processes may converge to sustain chronic pelvic pain despite the absence of an ongoing infection [13]. Hypertonicity of the pelvic floor muscle, dysfunctional myofascial trigger points [14,15] and alterations in urogenital microbiota composition further elucidate the mechanisms of CP/CPPS [16,17]. Dysbiosis of the gut microbiota has been increasingly implicated in the pathophysiology of CP/CPPS through a coordinated brain–gut–prostate axis. Alterations in microbial composition can increase the production of microbial-derived components, such as lipopolysaccharides (LPS), and modify metabolite profiles, including short-chain fatty acids, which collectively disrupt intestinal barrier integrity and promote systemic inflammation. While studies in benign prostatic hyperplasia (BPH) and prostate cancer have demonstrated elevated inflammatory mediators such as IL-6 and IL-18 associated with gut microbial imbalance [18,19], similar mechanisms are hypothesized to contribute to CP/CPPS, although direct clinical evidence remains limited.

Diagnosis of CP/CPPS depends on duration of symptoms (≥3 months), systematicclinical evaluation, and exclusion of anatomical or infectious conditions. However, proper diagnosis of CP/CPPS and its differential diagnosis for associated entities could be a challenge requiring follow-up of proposed diagnostic algorithms [20].

Anxiety and depression frequently co-occur in CP/CPPS, reflecting overlapping yet partially distinct neurobiological mechanisms that interact with chronic pelvic pain and immune alterations. Shared pathways include neuroinflammation, HPA axis dysregulation, and alterations in monoaminergic neurotransmission. Anxiety tends to involve hyperarousal and heightened amygdala-driven threat processing, whereas depression is characterized by anhedonia, dopaminergic reward dysfunction, and deficits in neuro-plasticity. Chronic pelvic pain, local immune perturbations, and persistent stress responses can exacerbate these affective symptoms, highlighting the bidirectional interactions between somatic and emotional mechanisms in CP/CPPS.

The “mechanistic matrix” of CP/CPPS classically includes (i) peripheral inflammation, (ii) central sensitization and (iii) catastrophizing as reviewed previously [21,22,23]. Recent evidence shifts and extends these mechanisms toward (iv) neurobiological maladaptation’s and neuroinflammation [24,25,26,27,28,29,30,31,32,33,34], especially in terms of anxiety and depression.

In this context, the mechanisms to be discussed in subsequent sections include: activation of microglia and astrocytes with the effects of proinflammatory cytokines (Section 2.1), neurochemical dysregulation (hormones, neurotransmitters) (Section 2.2), HPA axis and stress response (Section 2.3), brain regions and painemotion interactions (Section 2.4), central sensitization and pain-emotion network connections, (Section 2.5) and psychosocial mechanisms with biopsychosocial interactions (Section 2.6).

### 2.1. Activation of Microglia and Astrocytes: The Effects of Proinflammatory Cytokines

CP/CPPS is associated with dysregulated immune responses involving hematopoietic-derived cells. Increased numbers of leukocytes have been identified in expressed prostatic secretions, semen, and post-massage urine, particularly in inflammatory (NIH category IIIa) disease [35,36]. These immune populations include CD4^+^ and CD8^+^ T lymphocytes, macrophages, and mast cells, which are key mediators of local inflammatory responses [16]. These infiltrating immune cells contribute to a pro-inflammatory microenvironment characterized by elevated levels of cytokines such as interleukin-6 (IL-6), tumor necrosis factor-alpha (TNF-α), and interleukin-1β (IL-1β) [37,38]. Such cytokine profiles have been consistently detected in prostatic secretions and semen of CP/CPPS patients and are implicated in nociceptor sensitization and peripheral pain signaling [16,39].

**Figure 1:**
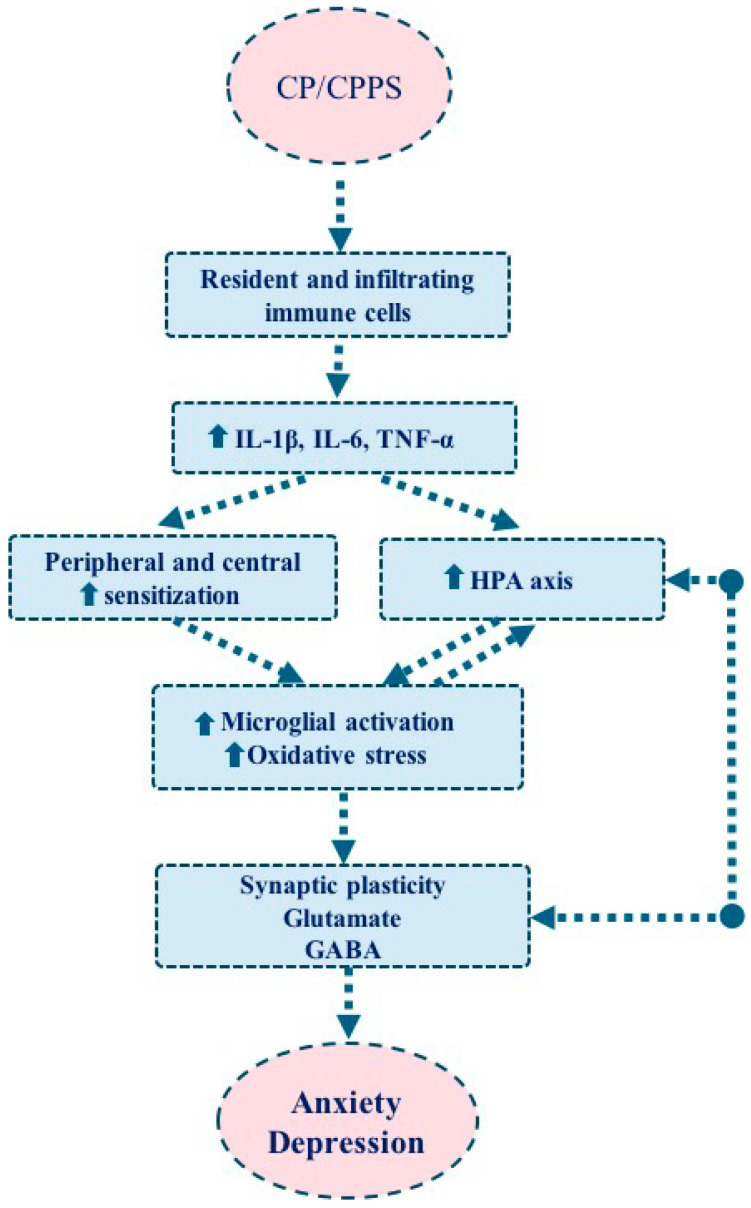
Proposed mechanistic cascade linking CP/CPPS to anxiety and depression. A multilevel mechanistic cascade initiated by CP/CPPS, in which prostatic inflammation activates resident and infiltrating immune cells (macrophages, mast cells, and T lymphocytes) leading to the release of pro-inflammatory cytokines (e.g., IL-1, IL-6, TNF). These peripheral immune processes drive activation of the HPA axis and promote both peripheral and central sensitization. Sustained inflammatory signaling and stress responses induce oxidative stress and activate glial cells, particularly microglia and astrocytes, which play a central role in maintaining neuroinflammation. This results in altered synaptic plasticity and neurochemical imbalance within pain–emotion circuits, ultimately contributing to anxiety and depression.

Inflammation in prostate tissue is accompanied by activation of microglia and astrocytes, which modulate neuronal function and play a critical role in maintaining neuroimmune homeostasis [40,41,42,43]. Actually, glial cells are believed to play a central role in chronic neuroinflammatory activity in peripheral and central nervous systems observed in CP/CPPS. Activated microglia release pro-inflammatory cytokines such as IL-1β, IL-6, and TNF-α, as well as neurotrophic factors that regulate synaptic plasticity. This process in-creases the sensitivity of peripheral sensory neurons and leads to hyperexcitability in central circuits [23,44]. Astrocytes maintain synaptic neurotransmission by regulating glutamate homeostasis, but the persistence of an inflammatory environment causes astrocytes to reinforce neuronal hyperexcitability [23,45,46]. Experimental animal models revealed a direct link between glial activation and pain behaviors such as mechanical allodynia and hyperalgesia. In autoimmune prostatitis models, microglial activation increases pain sensitivity through the upregulation of purinergic receptor P2X, ligand-gated ion channel 4 (P2X4) receptors and brain-derived neurotrophic factor (BDNF), while astrocyte activation shows a similar effect via Connexin 43 (Cx43) and C-X-C motif chemokine ligand 1 (CXCL1) pathways [41,42]. The effects of these processes on both pain and mood are summarized in Figure 1.

It is important to emphasize that both glial cell types can influence both pain and mood changes in central circuits and this effect may vary due to individual variations. These neuroimmune interactions and their clinical consequences are summarized in Table 1.

Pro-inflammatory cytokines (IL-1β, IL-6, TNF-α) contribute to a self-reinforcing cycle in CP/CPPS. Peripheral prostatitis induces inflammatory signaling that can activate glial cells and sensitize neurons, promoting chronic pain [48,49]. In fact, peripheral inflammation within the prostate is considered the primary initiating event, driven by activation of resident and infiltrating immune cells, including macrophages, mast cells, and T lymphocytes, which release pro-inflammatory cytokines (Figure 1) [40,50,51]. On the other hand, these mediators sensitize peripheral nociceptors and enhance afferent signaling to the central nervous system [50]. Sustained input leads to activation of spinal cord glial cells, including microglia and astrocytes [40,51]. As illustrated in Figure 1, CP/CPPS can be conceptualized as a multi-level mechanistic cascade in which peripheral inflammation initiates neuroimmune and neuroendocrine responses that progressively engage central sensitization and brain circuit dysfunction. Experimental evidence from central nervous system models further shows that microglia-derived IL-6 triggers astrocyte apoptosis in the hippocampus, leading to depression-like behavior [52], while IL-6 can also disrupt synaptic transmission and cognitive/emotional regulation via glutamate-dependent mechanisms [53,54]. Moreover, IL-6 signaling in the hypothalamus modulates HPA axis activity and cortisol release, strengthening the link between chronic inflammation and psychiatric symptoms [55]. TNF receptor signaling may additionally influence microglial activation and neuronal excitability, potentially contributing to this neuroimmune feedback loop [56]. Collectively, these findings indicate that glial cells and cytokines play dual roles in both pain and psychiatric comorbidities.

It should be noted that TNF-α can exert both pro-inflammatory and neuroprotective effects in the central nervous system through both TNFR1 and TNFR2 pathways [57]. TNF-α has been shown to enhance long-term potentiation in dorsal root-spinal cord circuits by reducing GABAergic inhibition and increasing NMDA activity, thus contributing to the maintenance of chronic pain. Microglia-derived TNF-α’s influence on synaptic transmission via extracellular vesicles may affect pain and emotional regulation at a central level [16,58]. However, individual heterogeneity and variability in peripheral-central interactions still complicate the precise clinical implications of this mechanism.

Several limitations should be considered when interpreting these findings. Much of the evidence linking glial activation to pain hypersensitivity in CP/CPPS originates from experimental animal models, which may not fully capture the biological and clinical heterogeneity of the human condition. Moreover, it remains unclear whether microglial and astrocytic activation represents a primary driver of chronic pain or a secondary response to persistent peripheral inflammation. Conflicting results also arise from cytokine signaling studies, as mediators such as TNF-α and IL-6 demonstrate context-dependent effects, exerting both pro-inflammatory and potentially neuroprotective actions depending on receptor subtype, cellular source, and timing of activation. Additionally, several mechanistic insights are derived from general neuroinflammation or depression models rather than CP/CPPS-specific research, limiting their direct translational relevance. Finally, substantial interindividual variability in neuroimmune signaling and peripheral–central interactions further complicate the interpretation and clinical applicability of these mechanisms.

In summary, we can state that microglia and astrocyte activation are not peripheral players in the pathophysiology of CP/CPPS. They play a central role in a complex mechanism that sustains chronic pain and psychiatric symptoms, along with peripheral inflammation, HPA axis, and central circuit interactions (Figure 1). Alongside glial activation, IL-1β, IL-6, and TNF-α appear to play a central role in the maintenance of chronic pain and psychiatric symptoms. However, the specific contributions of cytokines to central-peripheral interactions, and individual differences remain under investigation. Therefore, targeting these cytokines offers a potentially promising approach in the treatment of both nociceptive and psychiatric symptoms.

### 2.2. Neurochemical Dysregulation in CP/CPPS: Mechanisms and Clinical Implications

#### 2.2.1. Neuroendocrine Dysregulation: Role of Hormonal and Neurotransmitter Alterations in CP/CPPS

**Figure 2:**
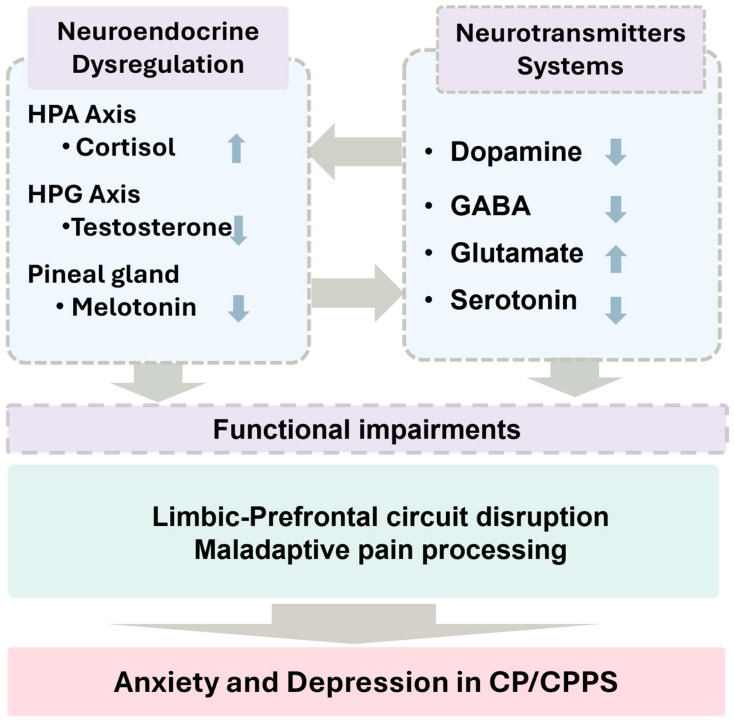
Neuroendocrine and neurotransmitter dysregulation underlying pain, anxiety, and depression in CP/CPPS. Figure depicts key neurochemical systems implicated in CP/CPPS, including dopaminergic, GABAergic, glutamatergic and serotonergic pathways, alongside endocrine modulators such as testosterone, melatonin, and adrenocortical hormones. Disruptions in these systems lead to impaired limbic-prefrontal circuit disruption and maladaptive pain processing. The figure emphasizes the hierarchical and interconnected nature of neurochemical dysregulation.

It has been increasingly recognized that neuroendocrine dysregulation interacts with neurotransmitter changes to exacerbate nociceptive signaling and affective disturbances in CP/CPPS (Figure 2). Hormonal imbalances and autonomic nervous system dysfunction play a crucial role in the maintenance of chronic pain.

Hormone-mediated signaling pathways and neuroendocrine mechanisms could be affected by increased oxidative stress and neuroinflammation observed in CP/CPPS which can ultimately lead to increased nociceptive sensitivity.

Importantly, these neurochemical alterations should be interpreted in the context of stress-system dysregulation (Section 2.3) via limbic-prefrontal circuit disruption and central pain–emotion circuits and maladaptive pain processing (Section 2.4), which together form an integrated pathophysiological framework for anxiety and depression in CP/CPPS. Figure 2 summarizes the core neuroendocrine and neurotransmitter mechanisms underlying CP/CPPS, emphasizing the hierarchical organization of key systems, including dopaminergic, serotonergic, glutamatergic, and GABAergic pathways. These systems interact with endocrine regulators such as testosterone and adrenocortical hormones, forming interconnected networks that modulate both nociceptive processing and affective states.

##### Core Neurotransmitter Mechanisms

Dopamine, serotonin, glutamate, and GABA represent the principal neurochemical systems underlying pain–emotion interactions in CP/CPPS.

Dopamine

Dopamine—mediated signaling has been identified as important endocrine factor in development of CP/CPPS symptomatology. Dysregulation of dopaminergic signaling within the mesolimbic networks, specifically the ventral tegmental area, nucleus accumbens, and prefrontal cortex attenuate reward processing while it increases the salience of nociceptive inputs what promote anhedonia, anxiety, and depression [59,60]. Dopaminergic deficiency also interacts with HPA axis hyperactivity and neuroinflammatory processes, reinforcing the pain–emotion loop [30,61]. Moreover, dopamine synthesis and transmission could be further impaired by CP/CPPS—induced neuroinflammatory processes, thus contributing to the central sensitization and maladaptive stress responses. The last one being the base for intensified pain perception and psychological burden [62]. Therefore, preclinical and clinical studies indicate that restoring dopaminergic tone ameliorates both pain perception and mood-related disturbances, confirming that dopamine system is one of the central factors in chronic pain and affective comorbidities related to CP/CPPS [59,60].

The affective dimension of chronic pain conditions, including CP/CPPS could be modulated by reward circuitry. Namely, pain relief activates mesolimbic reward pathways, particularly dopaminergic signaling between the ventral tegmental area and nucleus accumbens, which provides a strong reinforcement signal [63]. Actually, dopaminergic D2 receptor signaling has been shown to mediate pain–reward circuits [64]. Likewise animal models of neuropathic pain demonstrated increased serotonergic and mesolimbic dopaminergic transmission, neurochemical alterations that are also implicated in affective disorders [65].

These dopaminergic mechanisms directly correspond to functional alterations in limbic–prefrontal circuits described in Section 2.4, highlighting the role of reward-processing dysfunction in pain chronification.

Serotonin

Reduced serotonergic tone impairs descending pain inhibition and contributes to anxiety and depression. Altered tryptophan metabolism and serotonin availability are consistently observed in CP/CPPS models and patients [66]. This mechanism is further elaborated through the kynurenine pathway in Section 2.2.2.

Glutamate/GABA Balance

Reductions in GABAergic inhibition, evidenced by decreased parvalbumin-positive interneurons, amplify limbic excitability, predisposing individuals to anxiety, depression, and intensified pain perception [49]. In addition, Šutulović et al. (2021) reported reduced hippocampal PV^+^ interneuron counts, which were associated with anxiety-like behaviors [67]. Glutamatergic hyperactivity also contributes to central sensitization and affective dysregulation [68,69]. Studies indicate that chronic pain is associated with an imbalance between glutamate and GABA within cortical networks, driving persistent hyperexcitability and dysfunctional descending modulation [70]. In parallel, neuroinflammatory activation of the cystine/glutamate antiporter system (system xc−) elevates extracellular glutamate, amplifying excitotoxicity and sustaining neuropathic pain [23,71]. These processes are further exacerbated by chronic stress and dysregulated tryptophan metabolism via the kynurenine pathway, which together enhance glutamatergic transmission and reinforce maladaptive pain circuits [71].

These neurotransmitter alterations represent core mechanisms underlying circuit-level dysfunction in the ACC, insula, and prefrontal cortex (Section 2.4), and are strongly influenced by stress-related HPA axis activity (Section 2.3).

##### Endocrine Modulators of Core Neurotransmitter Systems

Endocrine factors are important players in the development of CP/CPPS and brain-related comorbidities. Current literature findings emphasize the role of testosterone, adrenocortical hormones and melatonin.

Testosterone

Reduced androgen levels enhance dorsal root ganglion excitability and spinal nociceptive transmission while simultaneously impairing control over limbic-prefrontal networks, including the prefrontal cortex, amygdala, and hippocampus [72,73]. Consistent with this mechanism, a testosterone-dependent modulation of inhibitory pathways and associated brain networks has been demonstrated in Sprague–Dawley rats [74]. Consequently, low testosterone predisposes patients to anxiety and depressive symptoms and promotes maladaptive pain processing, particularly when shifts in estrogen/testosterone ratios amplify peripheral inflammation.

Clinical evidence supports this mechanism. Lee and Lee (2016) reported that men with lower testosterone levels exhibit significantly greater CP/CPPS symptom severity [32]. Experimental models further confirm depression-like behaviors and cognitive deficits associated with disrupted neuroendocrine signaling [9]. Endocrine research has demonstrated that testosterone deficiency and hypothalamic–pituitary—gonadal (HPG) axis dysregulation increase the risk of depression, with genetic studies revealing a link between testosterone levels and major depressive disorder [75,76]. Findings by Hrnčić et al. (2025) suggest that CP/CPPS-related neuroendocrine alterations may interact with hippocampal mechanisms contributing to depressive-like pathology [77].

These findings also indicate that androgen deficiency may indirectly modulate neurotransmitter systems (e.g., dopaminergic and serotonergic signaling) and interact with HPA axis activity, further contributing to central sensitization (Section 2.2 and Section 2.3).

Adrenocortical Hormones

In men with CP/CPPS, levels of corticosterone, aldosterone and progesterone are significantly altered, and these hormonal alterations correlate with the symptom severity [78].

In parallel, metabolomic profiling in CP/CPPS patients reveals dysregulation of pathways related to inflammation, oxidative stress, energy metabolism, and neurotransmitter precursors [79]. These hormonal imbalances may modulate central serotonergic pathways, given that sex hormones can strongly influence tryptophan/serotonin metabolism. Altered serotonin metabolism and tryptophan catabolism have been found in rats with CP/CPPS [80], which has been further discussed in Section 2.2.2. Estrogen-dependent modulation of serotonergic signaling has been demonstrated to influence nociceptive sensitivity in animal models [81]. Moreover, gut–brain–microbiome interactions further shape serotoninergic regulation and may contribute to emotional vulnerability [82].

Importantly, these endocrine alterations are closely linked to HPA axis dysregulation and chronic stress responses (Section 2.3), further reinforcing neurochemical imbalance and central sensitization.

Melatonin

As a regulator of circadian rhythms and oxidative stress, melatonin deficiency disrupts sleep architecture, amplifies pain sensitivity, and reduces endogenous anti-inflammatory defenses. Experimental prostatitis models demonstrate that melatonin supplementation attenuates prostatic inflammation and pelvic pain via Sirt1-dependent inhibition of the NLRP3 inflammasome [83]. Extending these findings, Wang et al. (2024) confirmed melatonin’s role in mitigating oxidative stress and inflammatory responses in CP/CPPS [84]. Importantly, CP/CPPS models also exhibit depression-like behaviors and cognitive impairment [9], while psychiatric research consistently reports reduced melatonin levels in patients with mood disorders [85]. These observations underline melatonin’s dual therapeutic potential due to its antioxidative and anti-inflammatory properties. Hence, melatonin can modulate neural pathways associated with chronic pain and affective disorders, such as anxiety and depression [86].

Although melatonin is not a primary driver of nociceptive signaling, it acts as an important modulatory factor linking sleep disturbances, oxidative stress, and neuroendocrine imbalance (see also Section 2.6).

These findings indicate that CP/CPPS involves a complex interplay between hormonal dysregulation, neurotransmitter imbalances, and neuroimmune alterations. Deficiencies in testosterone, melatonin, and adrenocortical hormones, alongside disrupted dopaminergic, serotonergic, GABAergic, and glutamatergic signaling, destabilize limbic-prefrontal circuits and promote central sensitization. This convergence increases vulnerability to anxiety- and depression-like behaviors as well as maladaptive pain processing (Figure 2). Sex-hormone–dependent modulation of serotonergic pathways and gut–brain–microbiome interactions further influence nociceptive and affective outcomes. These insights highlight potential therapeutic targets, including restoration of endocrine balance, modulation of neurotransmitter systems, and integrated interventions addressing both cognitive-emotional and somatic dimensions of CP/CPPS. As illustrated in Figure 2, neuroendocrine and neurotransmitter dysregulation contribute to pain, anxiety, and depression in CP/CPPS.

#### 2.2.2. Tryptophan–Kynurenine Pathway in Pain and Psychiatric Comorbidities in CP/CPPS

The tryptophan–kynurenine pathway represents an important link between immune activation and central neurotransmitter balance. Indoleamine-2,3-dioxygenase (IDO), the rate-limiting enzyme of this pathway, is upregulated under inflammatory conditions and diverts tryptophan from serotonin synthesis toward kynurenine metabolites [87,88]. Notably, it is of interest for us that disruption of the tryptophan–kynurenine pathway reduces serotonergic tone, which could lead to increased pain sensitivity, as well as increased depression and anxiety susceptibility in CP/CPPS [89]. Indeed, changes in serotonin/tryptophan ratios have been clinically observed in expressed prostatic secretions and plasma of CP/CPPS patients [90]. This is predominantly mediated by activation of IDO. Increased IDO activity and elevated kynurenine levels correlate strongly with depressive and anxiety symptoms, cognitive impairment, and reduced neuropsychological performance, alongside amplified pain perception as demonstrated in clinical studies [88,89].

Under inflammatory conditions, such as CP/CPPS, pro-inflammatory cytokines upregulate IDO and diverts tryptophan from serotonin synthesis into the kynurenine pathway. This reduces central serotonergic tone and increases neuroactive metabolites implicated in neurotoxicity, neuroinflammation, and central sensitization [91]. This has been proven in animal models of CP/CPPS [92]. Such bidirectional interactions between immune signaling and tryptophan metabolism have been highlighted across different chronic pain disorders [93,94].

Preclinical studies showed that psychological stress enhances IDO1 activity and accelerates kynurenine pathway flux, producing immunosuppressive and neuromodulatory effects that contribute to central sensitization and affective dysregulation [95,96,97].

Jointly, these data support the concept that dysregulated tryptophan metabolism, driven by cytokine activity, stress physiology, and neuroimmune crosstalk, constitutes a mechanistic link connecting chronic pain, cognitive symptoms, and psychiatric vulnerability in conditions such as CP/CPPS. Therefore, it could be suggested that pharmacological modulation of this pathway or its modulation by herbal and immunomodulatory strategies may offer therapeutic value [98,99,100].

Key neurochemical and hormonal alterations implicated in CP/CPPS and their clinical consequences are summarized in Table 2.

#### 2.2.3. CO-Mediated Signaling in the Mechanism of CP/CPPS

Carbon monoxide (CO) is one of the gaseous neurotransmitters with anti-inflammatory and neuromodulatory properties [101,102]. Endogenously produced at low concentrations, CO exerts potent antioxidant effects, regulates HPA axis activity, and preserves parvalbumin-positive interneurons within hippocampal and limbic circuits. Controlled pharmacological delivery via slow-release CO donors such as CORM-A1 allows precise modulation of these processes, offering a strategy to simultaneously address nociceptive and affective dysregulation.

CO exerts anti-inflammatory effects primarily through the mitogen-activated protein kinase (MAPK) pathway. It inhibits the production of pro-inflammatory cytokines and reduces leukocyte infiltration in experimental models [101]. Additionally, CO reduces oxidative stress, limiting reactive oxygen species (ROS) accumulation and protecting tissues from inflammation-induced damage [102]. Since oxidative stress and inflammation are known contributors to both pain and mood disorders, CO’s ability to modulate these pathways may directly influence anxiety- and depression-like behaviors observed in CP/CPPS [9,67]. Administration of CO-releasing molecules (CORMs), such as CORM-A1, has been shown to reduce both pain and anxiety-related behaviors, suggesting that CO signaling can modulate the neurobiological substrate [103]. Mechanistically, CO’s antioxidant and anti-inflammatory effects in the brain may protect hippocampal neurons and interneurons, restoring normal circuitry implicated in emotional regulation. Beyond mood regulation, CO may influence neuronal excitability. Slow-releasing CO donors were found to modulate neural excitability in CP/CPPS models, likely via stabilization of neuronal networks disrupted by chronic inflammation and oxidative stress [104]. These findings suggest that CO can protect the central nervous system from inflammation-driven hyperexcitability, which may contribute to both pain perception and affective symptoms. These mechanisms are summarized in Table 3, highlighting the molecular and behavioral relevance of CO signaling in CP/CPPS models. The evidence positions CO as a mediator linking chronic pelvic inflammation to emotional and cognitive comorbidities, supporting its therapeutic potential for alleviating pain as well as anxiety and depression in CP/CPPS.

### 2.3. HPA Axis and Stress Response

The central pathogenic factor in anxiety is frequently proposed to be chronic stress acting through the sustained activation of the HPA axis. HPA is a major stress system in the body in which corticotropin-realizing hormone (CRH) from the hypothalamus acts on the anterior pituitary gland to produce adrenocorticotropine hormone (ACTH), which elicit cortisol release from adrenal cortex.

The HPA axis represents a central integrative system linking neuroendocrine, neurochemical, and psychosocial mechanisms in CP/CPPS (see Section 2.2 and Section 2.6). Rather than acting in isolation, HPA dysregulation both influences and is influenced by neurotransmitter systems, inflammatory processes, and chronic pain signaling.

Findings suggest that prolonged glucocorticoid (cortisol) elevation leads to structural and functional changes in limbic regions, particularly the amygdala and hippocampus. It is known that chronic hypercortisolemia is associated with reduced dendritic branching, suppression of neurogenesis in the dentate gyrus and even neural atrophy. These structural alterations are accompanied by deterioration in declarative memory, contextual processing and cognitive flexibility. In contrast, the effects of sustained elevated cortisol on amygdala demonstrate an opposite effect, resulting in dendritic hyperthopy and increased arborization. This remodeling in amigdala has been linked to enhanced emotional salience processing and increased anxity-like behavior. These effects were elaborated in more detail previously [105,106].

These region-specific effects of cortisol directly correspond to alterations in limbic–prefrontal circuits described in Section 2.4, particularly involving the amygdala, hippocampus, and prefrontal cortex, which together regulate pain perception and emotional responses.

On the other hand, prolonged HPA activation may be compensationally linked to hypocortisolemia. From an evolutionary standpoint, these alterations may represent adaptive trade-offs prioritizing rapid threat detection and emotional learning (amygdala-dependent processes) over detailed contextual memory formation (hippocampal dependent processes) in environments characterized by sustained threat, which aligns with the allostatic load framework [107,108,109].

Clinical studies have shown a correlation between HPA axis dysfunction and stress response, pain intensity, and quality of life in CP/CPPS patients. However, these data are heterogeneous and methodologically limited [110,111]. Although impaired cortisol response has been associated with anxiety and depression scores, this association alone may be insufficient to explain symptom severity.

This is consistent with the concept that HPA axis dysfunction acts synergistically with neurochemical dysregulation (Section 2.2.1), including alterations in serotonergic, dopaminergic, and glutamatergic signaling, which together drive central sensitization.

Additionally, impaired HPA activity contributes to neuronal damage through oxidative stress, mitochondrial dysfunction, and neuroinflammation [108,110]. These processes impair the prefrontal cortex’s capacity to modulate pain, while increased activity in the amygdala and hippocampus intensifies emotional responses such as fear, anxiety, and depression [112,113]. Such changes reflect a breakdown in top-down inhibitory control and enhanced bottom-up emotional reactivity, which are key features of maladaptive pain–emotion circuitry in CP/CPPS (Section 2.4). A critical point of debate here is whether changes in these circuits are directly related to HPA dysfunction or to peripheral inflammation, variations in the immune system, and life experiences. Current data suggest that these factors likely act in conjunction, but the precise mechanism remains unclear.

Psychosocial stressors such as pain catastrophizing, sleep disturbances, and reduced social support (Section 2.6) can further amplify HPA axis activation, thereby reinforcing neuroendocrine and neurochemical dysregulation.

Clinical studies suggest that interventions aimed at normalizing HPA axis activity, like cognitive-behavioral therapy, mindfulness-based practices, chronic stress management, and pharmacological modulation, offer significant potential in managing both pain and psychiatric symptoms [91,92,93]. However, the effectiveness of these interventions may vary due to individual differences and the heterogeneity of HPA-psychiatric circuit interaction. Therefore, the HPA axis should be viewed as a key mediator translating psychosocial stress into neurobiological changes that sustain chronic pain and affective disturbances in CP/CPPS.

**Figure 3:**
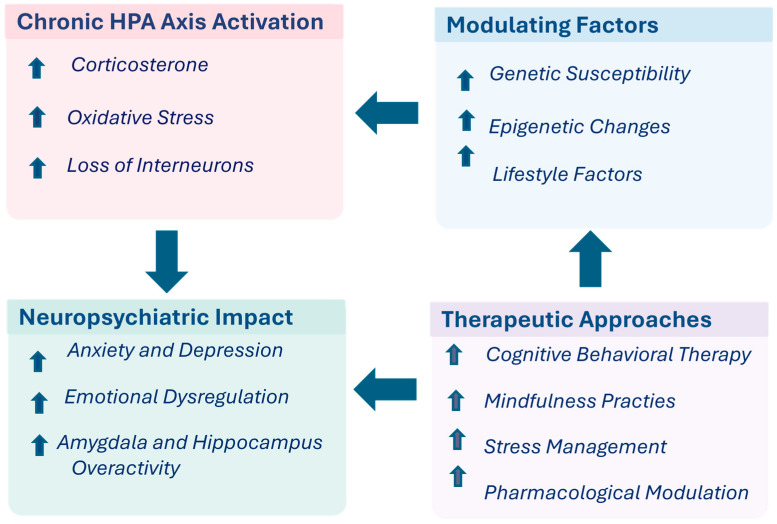
Chronic HPA Axis Activation in CP/CPPS: mechanisms, modulating factors, and neuropsychiatric implications. Figure illustrates chronic activation of the hypothalamic–pituitary–adrenal (HPA) axis as a central mechanism in CP/CPPS, characterized by increased corticosterone levels, elevated oxidative stress, and loss of inhibitory interneurons. These changes contribute to neuropsychiatric outcomes, including increased anxiety and depression, emotional dysregulation, and hyperactivity of limbic regions such as the amygdala and hippocampus. HPA axis activity is further shaped by modulating factors, including genetic susceptibility, epigenetic modifications, and lifestyle influences, highlighting inter-individual variability in stress responses. Therapeutic approaches—including cognitive-behavioral therapy, mindfulness-based practices, stress management and pharmacological modulation—target both neuropsychiatric outcomes and upstream modulatory factors. Overall, the model emphasizes bidirectional interactions and supports the need for future multidimensional approaches integrating neuroendocrine, environmental, genetic, and immune factors in CP/CPPS.

Future research should focus on developing multidimensional models that take into account HPA dysfunction along with environmental, genetic, and immune factors. The integrative effects of HPA axis dysregulation on stress response, modulatory factors, and neuropsychiatric outcomes in CP/CPPS are summarized in Figure 3.

### 2.4. Brain Regions and Pain—Emotion Interactions in CP/CPPS

Certain brain regions and neural networks are morphological substrates for pain emotion interactions. Namely, neuroimaging studies consistently identify the anterior cingulate cortex (ACC), insula, prefrontal cortex (PFC), amygdala, and hippocampus as key regions involved in maladaptive behavioral response to pain. These brain circuits represent the central integrative framework through which neurochemical (Section 2.2), neuroendocrine (Section 2.3), and psychosocial (Section 2.6) factors converge in CP/CPPS.

A large proportion of neuroimaging studies indicated that CP/CPPS is associated with extensive reorganization of these central nervous system networks that integrate nociceptive and emotional processing. Functional MRI studies report that CP/CPPS patients show increased resting-state Degree Centrality (DC) in the right ACC, bilateral insula, left amygdala, and right middle frontal cortex compared with healthy controls. The finding that ACC centrality closely correlates with National Institutes of Health Chronic Prostatitis Symptom Index (NIH-CPSI) symptom severity is particularly interesting.

This supports the concept that the ACC functions as a central hub integrating nociceptive input with affective and cognitive processing, thereby linking symptom severity with underlying network dysfunction. Having these in mind, it could be suggested that the alterations in ACC function are marker of disease burden and serve as a central hub for integrating nociceptive and affective signals [31].

Neuroimaging studies also pointed out some important morphological alterations in these brain regions. Voxel-based morphometry (VBM) and Diffusion Tensor Imaging (DTI) analyses reveal reduced grey matter volume in the ACC and insula in CP/CPPS, alongside compromised white matter integrity in tracts connecting sensorimotor and limbic regions. Additionally, gray matter reductions in the ACC and insula correlate with symptom duration, suggesting progressive neuroplastic adaptations to chronic pelvic pain [27,29]. These structural changes are consistent with chronic exposure to stress hormones (Section 2.3) and persistent neurotransmitter imbalance (Section 2.2), reinforcing the concept of activity-dependent neuroplasticity in CP/CPPS.

White matter analyses from the Multidisciplinary Approach to the Study of Chronic Pelvic Pain (MAPP II) cohort further highlight increased mean diffusivity in tracts linking the postcentral gyrus and precuneus, as well as decreased structural connectivity between the cuneus and cingulate cortex, reflecting maladaptive network remodeling that may perpetuate pain and emotional dysregulation [114,115].

The anterior insula exhibits hyperactivation in CP/CPPS, which is strongly associated with pain intensity and negative affect [27,29]. Alterations in the medial and dorsolateral PFC suggest impaired descending control over limbic pathways, leading to decreased cognitive modulation of pain and emotion. Functional and perfusion changes in these prefrontal regions likely contribute to increased pain sensitivity, catastrophizing, and comorbid anxiety and depression observed in CP/CPPS populations [116], with these findings supported by human studies [116]. These findings directly correspond to neurochemical mechanisms described in Section 2.2, particularly reduced GABAergic inhibition and increased glutamatergic activity, which impair top-down regulatory control.

The hippocampus, critical for contextual memory, stress regulation, and emotional processing, also shows morphological and functional alterations in CP/CPPS. Specifically, animal models of prostatitis demonstrated parvalbumin-positive interneuron loss, increased oxidative stress, and HPA axis hyperactivity in the hippocampus, correlating with anxiety- and depression-like behaviors [47,67]. This provides a direct mechanistic link between HPA axis dysregulation (Section 2.3), neuroinflammation, and behavioral outcomes.

In humans, altered hippocampal connectivity with the posterior cingulate cortex is associated with elevated systemic IL-6 levels, linking neuroinflammation to dysregulated limbic circuits and affective disturbances [117,118]. Similarly, the amygdala exhibits hyperactivity and abnormal connectivity with the insula and PFC, amplifying fear, anxiety, and pain perception, and strengthening the bidirectional relationship between chronic nociceptive input and negative emotional states [119,120,121]. Amygdala hyperactivity, in particular, reflects increased emotional salience and is strongly influenced by chronic stress and cortisol exposure, further linking circuit dysfunction to HPA axis activity (Section 2.3).

Structural, functional, and neurochemical evidence indicates that CP/CPPS involves not only peripheral inflammatory processes but also extensive reorganization of central networks that integrate pain and emotions. Dysregulation within ACC-insula-amygdala-PFC circuits, together with hippocampal dysfunction and sustained neuroinflammation, appears to create a self-perpetuating loop in which chronic pelvic nociception exacerbates affective symptoms, and vice versa.

Within this framework, brain circuits act as the final common pathway through which endocrine, neurochemical, and psychosocial factors interact, reinforcing chronic pain and emotional dysregulation.

Accordingly, neuromodulation strategies, cognitive-behavioral therapy, and pharmacological interventions targeting neuroinflammation or GABAergic/cholinergic signaling may help restore network function and alleviate both pain and psychiatric comorbidities in CP/CPPS patients [122,123,124].

This integrative perspective supports the conceptualization of CP/CPPS as a disorder of maladaptive pain–emotion circuitry rather than a purely peripheral condition, aligning neurobiological findings with clinical and psychosocial observations (Section 2.6).

### 2.5. Central Sensitization and Pain-Emotion Network Connections

Central sensitization plays a critical role in the intensification of pain perception in CP/CPPS. The process of central sensitization refers to the chronic amplification of peripheral nociceptive stimuli at the spinal and supraspinal levels [15,39,116]. Central sensitization represents a key mechanistic bridge linking peripheral inflammation with neurochemical dysregulation (Section 2.2), HPA axis dysfunction (Section 2.3), and brain network alterations (Section 2.4).

In human studies, electrophysiological assessments have confirmed central sensitization in CP/CPPS patients, demonstrating increased spinal excitability and altered pain processing [125]. In parallel, animal models of experimental autoimmune prostatitis (EAP) have shown that peripheral inflammation can trigger central neurobehavioral changes, including microglial activation (increased Iba1), astrocyte reactivity (GFAP), elevated IL-1β and indolamine 2,3-dioxygenase (IDO) expression, decreased serotonin levels, and NF-κB pathway activation [126,127]. These findings suggest that peripheral inflammation can affect central neurobehavioral circuits and play a role in the emergence of depressive and anxiety-like behaviors.

Clinical investigations further support these findings, demonstrating significantly increased perceived stress, anxiety, and depression scores, along with an impaired cortisol awakening response in CP/CPPS patients [28]. Moreover, a meta-analysis of human studies further supports the concept of CP/CPPS as a psychoneuromuscular disorder characterized by strong interactions between stress-related mechanisms and chronic pain [128]. This aligns with the role of HPA axis dysregulation (Section 2.3), where chronic stress enhances central sensitization through sustained cortisol exposure, neuroinflammation, and altered neurotransmitter signaling. These findings reveal the existence of a chain of mechanisms extending from the animal model to the clinical picture, and a continuous interaction between peripheral inflammation and central emotional circuits.

At the circuit level (Section 2.4), central sensitization is reflected in hyperactivity of the ACC, insula, and amygdala, alongside impaired prefrontal inhibitory control, reinforcing the bidirectional relationship between pain and emotional processing.

Consequently, in CP/CPPS, central sensitivity and the pain-emotion network should be understood not merely as a symptomatic relationship, but as a holistic pathophysiological framework in which peripheral inflammation, HPA dysfunction, and oxidative stress converge. This approach is crucial for understanding both the mechanism and potential therapeutic targets.

### 2.6. Psychosocial Mechanisms in CP/CPPS: Biopsychosocial Interactions and Comprehensive Management

Persistent pelvic pain acts as both a physical and psychological stressor, activating emotional and physiological pathways that can lead to anxiety, depression, and reduced quality of life [10,124]. Population-level studies highlight the complex interplay between psychological mechanisms and symptom severity in men with CP/CPPS. Psychosocial factors should be viewed as active modulators of biological processes rather than merely secondary consequences, interacting directly with HPA axis activity (Section 2.3), neurochemical systems (Section 2.2), and brain circuits (Section 2.4).

Data from the NIH Chronic Prostatitis Cohort and the MAPP Research Network indicate that both pain and urinary symptoms are associated with long-term decline in physical and mental quality of life [129,130,131]. Certain urinary symptoms, such as frequency and incomplete emptying, may disrupt daily functioning, although pelvic pain remains the strongest individual predictor of impaired well-being [132,133,134,135]. Multidimensional interventions that address both somatic and psychological aspects including cognitive-behavioral therapy and stress management have been shown to improve quality of life in these patients [25].

Psychological factors include perceived stress, coping strategies, social support, and particularly pain catastrophizing. Hence, these factors play a key role in shaping patients’ experiences of the CP/CPPS.

Catastrophizing is especially maladaptive, as it amplifies pain perception, enhances emotional distress, and contributes to the development or exacerbation of anxiety and depressive symptoms [5,34]. Namely, early NIH CPCRN studies highlight the impact of catastrophizing: men who engage in more catastrophic thinking report higher pain intensity, greater functional limitations, more severe urinary symptoms, and elevated depressive symptoms, even after accounting demographic and psychosocial factors [34]. Neurobiologically, catastrophizing has been associated with increased activation of the anterior cingulate cortex and amygdala (Section 2.4), as well as enhanced HPA axis reactivity (Section 2.3), thereby linking cognitive-emotional processes with central pain modulation.

Helplessness during catastrophic episodes and low social support further diminish mental health–related quality of life [136]. Adolescents with prostatitis-like symptoms show similar patterns, with catastrophizing predicting poor quality of life independent of symptom severity. Long-term follow-up confirms that catastrophizing and related psychosocial factors influence pain trajectories and overall well-being over years [137].

Sexual dysfunction is a common and significant consequence of CP/CPPS. Studies indicate a higher prevalence of sexual problems in affected men, which are associated with both pain and psychological distress [138].

Sleep disturbances, particularly insomnia, also contribute substantially to reduced quality of life by exacerbating both somatic and psychological symptoms. Experimental and clinical studies suggest that poor sleep can trigger inflammatory responses, alter hormonal regulation, and increase oxidative stress, thereby amplifying pain sensitivity and urinary dysfunction [139]. Sleep apnea has been linked to the development of prostatitis, highlighting the multifactorial impact of sleep disorders on pelvic health [140]. Sleep disturbances further dysregulate melatonin signaling (Section 2.2), disrupt HPA axis rhythmicity (Section 2.3), and enhance central sensitization processes (Section 2.5), thereby reinforcing the pain–emotion cycle.

The bidirectional relationship between CP/CPPS and sleep, where urinary symptoms disrupt sleep and poor sleep, in turn, amplifies pain and psychological distress, underlines the importance of incorporating sleep management into comprehensive treatment plans [141,142].

In summary, men with CP/CPPS experience a complex interplay of somatic symptoms, psychological mechanisms, social support deficits, sexual dysfunction, and sleep disturbances. Within an integrated framework, these psychosocial factors interact with neurobiological mechanisms to sustain chronic pain, central sensitization, and affective comorbidities, forming a self-reinforcing biopsychosocial loop.

Effective management requires comprehensive, multivalent strategies, including cognitive-behavioral therapy, stress management, social support enhancement, sexual health interventions, sleep optimization, and complementary approaches such as neural therapy [12,142,143]. Addressing both biological and psychosocial dimensions can significantly improve symptom burden and overall quality of life in this population.

## 3. Limitation

Although human and animal studies have made significant contributions to understanding the neurobiological mechanisms of CP/CPPS, these studies exhibit some methodological and translational limitations.

A significant limitation in CP/CPPS research is clinical heterogeneity. When considering the pathophysiology of CP/CPPS, the role of psychological factors is too significant to ignore. Psychological factors accompanying pain duration and symptom severity can vary considerably among patients. Namely, it is not always clear whether functional or structural changes detected in the central nervous system directly reflect disease-specific biological mechanisms or are a consequence of the psychological burden accompanying chronic pain. However, many neurobiological studies in the literature either do not measure psychological variables in sufficient detail or do not systematically control them as confounding factors in the analyses. This deficiency indicates that caution is needed in interpreting the findings.

Another limiting factor is their cross-sectional design, which prevents us from determining whether the observed brain changes are cause of the disease or if they are secondary neuroplastic adaptations that occur as a result of chronic pain.

Research on neuroinflammatory mechanisms at the molecular level is important research area but still limited in number and sample size. Shoskes et al. (2021), in their NanoString-based analysis performed on 10 CP/CPPS patients and 7 controls, detected significant differences in neuroinflammation-related gene expression patterns in blood and urine [144]. Changes were reported particularly in gene sets associated with inflammation, cytokine signaling, neurogenic pain, and adaptive immunity. Furthermore, some specific expression patterns were identified according to UPOINT phenotype domains. However, due to the limited sample size and the cross-sectional design of the study, a direct relationship between these gene expression changes and the severity of clinical symptoms or their causal role in the pathogenesis of the disease cannot be clearly established.

Experimental CP/CPPS models are generally considered to have significant disease validity and high translational potential [93,94]. However, factors that could diminish cross-study comparability and translational validity are heterogeneity in model induction methods (autoimmune vs. chemically induced), variations in protocols, chemical agents used, as well as validation strategies in regard to detection of hyperalgesia and prostate inflammation, what has been recently reviewed in more details elsewhere [11].

## 4. Therapeutic Implications and Future Directions

The mechanistic insights reviewed herein highlight several potential therapeutic avenues for anxiety and depression in CP/CPPS. Targeting neuroinflammation, particularly through modulation of glial activity and pro-inflammatory cytokines, may reduce both pain sensitivity and affective symptoms. Similarly, restoring neurochemical balance via normalization of testosterone, melatonin, dopamine, serotonin, and GABAergic signaling could improve mood and cognitive outcomes while attenuating nociceptive processing. HPA axis modulation through behavioral interventions, stress management, and pharmacological approaches may further mitigate maladaptive stress responses and associated limbic-prefrontal dysregulation. Emerging strategies, including CO-mediated neuromodulation, tryptophan–kynurenine pathway modulation, and gut–brain–microbiome interventions, offer promising adjuncts to traditional therapies. Future research should focus on individualized, multimodal treatment strategies that integrate peripheral, central, and psychosocial targets, with attention to patient heterogeneity and longitudinal outcomes. Overall, translating these mechanistic insights into clinical practice may advance both symptom control and quality of life in CP/CPPS patients.

## 5. Conclusions

Chronic prostatitis/chronic pelvic pain syndrome (CP/CPPS) may represent more than a purely urological condition, encompassing dynamic alterations in central nervous system function and complex interactions among neuroimmune and neuroendocrine pathways. Evidence indicates that the association between chronic pelvic pain and comorbid anxiety or depression in CP/CPPS may be mediated by central sensitization, glial cell activation, pro-inflammatory cytokines, HPA axis dysregulation, and neurotransmitter imbalances.

Structural and functional imaging studies have reported changes in the anterior cingulate cortex, insula, amygdala, hippocampus, and prefrontal cortex, which may reflect maladaptive reorganization of networks involved in pain perception and mood regulation. In parallel, dopaminergic reward circuits, serotonergic signaling, tryptophan–kynurenine metabolism, and CO-mediated neuromodulatory mechanisms have been proposed to contribute to the persistence of pain and associated psychiatric symptoms. The interaction between these biological processes and psychosocial factors further underscores the multifactorial, biopsychosocial nature of CP/CPPS.

These observations collectively highlight the need for integrative research approaches that combine neuroimaging, neuroimmune biomarkers, metabolomics, and microbiome analyses to better characterize patient subgroups and inform personalized therapeutic strategies. Multidisciplinary interventions incorporating pharmacological, behavioral, and psychosocial components should be carefully evaluated to optimize long-term outcomes in CP/CPPS.

## Figures and Tables

**Table 1:** Glial Activation and Proinflammatory Cytokine Cascade in CP/CPPS.

Mechanism	Cellular Mediators	Molecular Outputs	Functional Impact	Clinical Manifestation	References
Peripheral inflammation	Prostatic immune cells	IL-1β, IL-6, TNF-α	Facilitates central neuroimmune signalling and primes glial cells	Initiation and maintenance of chronic pelvic pain; increased stress sensitivity	[39,40,41]
Microglial activation	Iba1^+^ microglia	Pro-inflammatory cytokines, BDNF, P2X4 receptor upregulation	Enhances neuronal excitability and central sensitization	Mechanical allodynia and hyperalgesia; affective vulnerability	[40,41,45]
Astrocyte reactivity	GFAP^+^ astrocytes	Pro-inflammatory mediators and altered glutamate homeostasis	Amplifies excitatory transmission and maintains inflammatory milieu	Pain amplification and emotional dysregulation	[42,45,47]
Cytokine cascade	IL-1β, IL-6, TNF-α	NF-κB, CREB pathway activation	Impairs synaptic plasticity and dendritic remodelling	Cognitive decline and affective instability	[43,44]
Synaptic remodelling	Microglia–astrocyte interaction	Reduced synaptic plasticity and excitatory–inhibitory imbalance	Impaired learning, memory, and mood regulation	Depression-like behaviour and stress susceptibility	[25,44]
Neuroimmune feedback loop	Glial–cytokine–neuron axis	Sustained neuroinflammatory signalling and neuronal hyperexcitability	Self-perpetuating cycle of pain and mood dysregulation	Chronic pelvic pain with anxiety/depression	[48,49,50,51]
Therapeutic modulation	Cytokine-targeted interventions (e.g., TNF-α and IL-6 modulation)	Reduction in neuroinflammatory signaling	Partial restoration of neuroimmune homeostasis	Improvement in nociceptive behaviours and anxiety-like symptoms	[44,52]

BDNF: Brain-Derived Neurotrophic Factor; CREB: cAMP Response Element-Binding Protein; GFAP: Glial Fibrillary Acidic Protein; IL-1β: Interleukin-1 Beta; IL-6: Interleukin-6; NF-κB: Nuclear Factor Kappa B; P2X4: P2X4 Purinergic Receptor; TNF-α: Tumor Necrosis Factor Alpha.

**Table 2:** Neurochemical and Hormonal Dysregulation in CP/CPPS: Mechanisms and Clinical Implications.

Neurochemical System/Pathway	Dysregulation in CP/CPPS	Mechanistic Effect	Clinical Implications	Key References
Serotonin (5-HT)	↓	Reduced serotonergic tone and altered nociceptive modulation	Anxiety, depression, increased pain sensitivity	[66,81,87,98]
Dopamine (VTA/NAcc)	↓	Impaired reward processing and mesolimbic dysfunction	Anhedonia, anxiety, depression	[62,63,64,86]
Norepinephrine (NE)	Dysregulated	Altered stress-related arousal and central pain modulation	Anxiety, pain hypersensitivity	[82,95,96]
GABA (PV^+^ interneurons)	↓	Reduced inhibitory control and limbic hyperexcitability	Anxiety, depression, amplified pain	[65,67]
Glutamate	↑	Increased excitatory transmission and central sensitization	Chronic pain, affective symptoms	[68,69,70,71]
Kynurenine pathway/ IDO	↑ flux	Shift toward neuroactive metabolites and reduced serotonin availability	Depression, anxiety, increased pain sensitivity	[91,94,97,100]
HPA axis/ Cortisol	↑	Dysregulated stress response and neuroendocrine imbalance	Anxiety, depression, stress sensitivity	[78,95,96]
Neuroactive steroids Melatonin	↓	Impaired circadian rhythm and reduced anti-inflammatory defense	Sleep disturbance, anxiety, depression	[83,84,85]

5-HT: Serotonin; GABA: Gamma-Aminobutyric Acid; HPA: Hypothalamic–Pituitary–Adrenal axis; IDO: Indoleamine-2,3-Dioxygenase; NAcc: Nucleus Accumbens; NE: Norepinephrine; PV^+^: Parvalbumin-Positive interneurons; VTA: Ventral Tegmental Area; ↓: decrease/downregulation; ↑: increase/upregulation.

**Table 3:** CO Signaling Pathways in CP/CPPS: Links to Pain, Anxiety, and Depression.

CO-Mediated Mechanism	Molecular/Cellular Effect	Observed Effect in CP/CPPS Models	Relevance to Anxiety/Depression	Key References
Anti-inflammatory signaling	Inhibition of pro-inflammatory cytokines via MAPK & NF-κB	Reduced pelvic inflammation and leukocyte infiltration	Chronic inflammation is linked to mood disorders; reducing cytokines may alleviate anxiety/depression	[101,102]
Oxidative stress reduction	Decreased ROS, protection of neurons and astrocytes	Lower oxidative damage in peripheral tissues and hippocampus	Oxidative stress in hippocampus contributes to depression- and anxiety-like behaviours	[9,67,102]
Neuromodulation of pain & mood	Restoration of hippocampal interneurons, astrocyte modulation	Attenuation of pain, anxiety- and depression-like behaviors	Supports emotional regulation circuits affected by chronic inflammation	[9,67,103]
Stabilization of neuronal excitability	Reduced seizure susceptibility	Protection of CNS circuits disrupted by inflammation	Neural network stabilization may reduce hyperexcitability linked to anxiety/depression	[104]

CNS: Central Nervous System; CP/CPPS: Chronic Prostatitis/Chronic Pelvic Pain Syndrome; MAPK: Mitogen-Activated Protein Kinase; NF-κB: Nuclear Factor Kappa B; ROS: Reactive Oxygen Species.

## Data Availability

No new data were created or analyzed in this study.
